# A Randomized Clinical Trial of Precision Prevention Materials Incorporating *MC1R* Genetic Risk to Improve Skin Cancer Prevention Activities Among Hispanics

**DOI:** 10.1158/2767-9764.CRC-21-0114

**Published:** 2022-01-11

**Authors:** John Charles A. Lacson, Scarlet H. Doyle, Jocelyn Del Rio, Stephanie M. Forgas, Rodrigo Carvajal, Guillermo Gonzalez-Calderon, Adriana Ramírez Feliciano, Youngchul Kim, Richard G. Roetzheim, Steven K. Sutton, Susan T. Vadaparampil, Brenda Soto-Torres, Peter A. Kanetsky

**Affiliations:** 1 Department of Cancer Epidemiology, H. Lee Moffitt Cancer Center & Research Institute, Tampa, Florida.; 2 Biostatistics and Bioinformatics Shared Resources, H. Lee Moffitt Cancer Center & Research Institute, Tampa, Florida.; 3 Public Health Program, Ponce Health Sciences University, Ponce, Puerto Rico.; 4 Department of Biostatistics and Bioinformatics, H. Lee Moffitt Cancer Center & Research Institute, Tampa, Florida.; 5 Department of Family Medicine, Morsani College of Medicine, University of South Florida, Tampa, Florida.; 6 Department of Health and Behavioral Outcomes, H. Lee Moffitt Cancer Center & Research Institute, Tampa, Florida.

## Abstract

**Purpose:**

Skin cancer incidence is increasing among Hispanics, who experience worse outcomes than non-Hispanic Whites. Precision prevention incorporating genetic testing for (melanocortin-1 receptor) *MC1R*, a skin cancer susceptibility marker, may improve prevention behavior.

**Experimental Design:**

Hispanic participants (*n* = 920) from Tampa, FL and Ponce, PR, were block-randomized within *MC1R* higher- and average-risk groups to precision prevention or generic prevention arms. We collected baseline information on demographics, family history of cancer, phenotypic characteristics, health literacy, health numeracy, and psychosocial measures. Participants reported weekday and weekend sun exposure (in hours), number of sunburns, frequency of five sun protection behaviors, intentional outdoor and indoor tanning, and skin examinations at baseline, 3 months, and 9 months. Participants also reported these outcomes for their eldest child ≤10 years old.

**Results:**

Among *MC1R* higher-risk participants, precision prevention increased sunscreen use (OR = 1.74, *P* = 0.03) and receipt of a clinical skin exam (OR = 6.51, *P* = 0.0006); and it decreased weekday sun exposure hours (β = −0.94, *P* = 0.005) and improved sun protection behaviors (β = 0.93, *P* = 0.02) in their children. There were no significant intervention effects among *MC1R* average-risk participants. The intervention did not elevate participant cancer worry. We also identified moderators of the intervention effect among both average- and higher-risk participants.

**Conclusions:**

Receipt of *MC1R* precision prevention materials improved some skin cancer prevention behaviors among higher-risk participants and their children and did not result in reduced prevention activities among average-risk participants. Despite these encouraging findings, levels of sun protection behaviors remained suboptimal among participants, warranting more awareness and prevention campaigns targeted to Hispanics

**Significance:**

Our results support a precision public health approach to reducing skin cancers among Hispanics, an underserved population in precision medicine, and may additionally improve preventive behaviors among their children.

## Introduction

Although the incidence of skin cancer—including basal cell carcinoma (BCC), squamous cell carcinoma (SCC), and melanoma—is lower among Hispanic individuals than among non-Hispanic White individuals, skin cancer rates have risen among Hispanics over the past several decades (1–3). In Puerto Rico, incidence of these three skin cancers increased over 300% between 1974 and 2005 (1). And in the United States, the incidence of melanoma increased an average of 0.6% annually between 2000 and 2018 among Hispanics of any race (3).

Hispanics also experience skin cancer health disparities and tend to be diagnosed with later stage melanoma and present with larger BCC and SCC, thus experiencing higher morbidity and mortality than non-Hispanic Whites (2, 4, 5). These disparities may arise due to lack of patient and clinician awareness about skin cancer risk in Hispanics and unequal access to health care (6, 7). The rapid growth of the Hispanic population in the United States will likely compound these trends (8).

The melanocortin-1 receptor (*MC1R*) gene is highly polymorphic and encodes a protein that is a primary regulator of skin pigmentation (9). Inherited variation at *MC1R* is strongly associated with increased risk for melanoma and non-melanoma skin cancers in populations of European ancestry, including among individuals who have limited phenotypic skin cancer risk characteristics (10–13). The proportion of melanoma risk attributable to carriage of the nine most common variants at *MC1R* approaches 45% (13, 14). Our pilot study showed that 56% of Hispanics in Puerto Rico and the Tampa Bay area—including individuals with diverse ancestry backgrounds—carry *MC1R* variants associated with elevated skin cancer risk, with *MC1R* minor allele frequencies ranging up to 10% (15). Thus, for a subset of Hispanics at increased genetic risk, precision prevention intervention incorporating *MC1R* genetic risk may improve preventive behaviors.

We conducted a randomized controlled trial among Hispanics in Tampa, FL and Ponce, PR, to examine whether providing precision prevention information communicating *MC1R* genetic risk improves primary and secondary skin cancer prevention activities compared with providing generic prevention information. On the basis of Protection Motivation Theory (16), we hypothesized detecting intervention effects among *MC1R* higher-risk participants, but not among average-risk participants. Finally, we assessed moderation of the intervention effect by baseline characteristics.

## Materials and Methods

### Participants and Setting

We recruited participants from eight primary care clinics and community health centers in Tampa, FL, and Ponce, PR between September 2018 and January 2020. For patients in Tampa with an upcoming clinic visit, we first obtained limited demographic information including ethnicity. Potentially eligible participants, that is, patients in Tampa for whom a non-Hispanic ethnicity was not noted in the clinic schedule (including those missing information on ethnicity) and all patients in Ponce, were approached in clinic and asked to complete a tablet-based screening questionnaire that solicited information on Hispanic ethnicity [Puerto Rican, Cuban, Dominican, Mexican/Mexican American/Chicano, Central or South American (other than Brazilian), other], race, history of skin cancer and skin examinations over the past year. Eligible participants self-identified as Hispanic and were at least 18 (Tampa) or 21 (Ponce) years of age. Exclusion criteria included having a skin examination within the past year, previous diagnosis of melanoma, and more than one previous diagnosis of BCC and/or SCC.

All participants provided written informed consent, and the study was conducted in accordance with the Declaration of Helsinki. The study was approved by the Institutional Review Boards of the University of South Florida (Tampa, FL; Pro00020044, approved August 30, 2018), Ponce Health Sciences University (Ponce, PR; 170807-BS, approved December 6, 2017), and the Comité de Seguimiento de la Investigación Clínica at Hospital Damas (HD 19-17, approved December 18, 2017). This trial was registered on clinicaltrials.gov (NCT03509467), and we followed CONSORT guidelines to report our trial design, analyses, and interpretation.

### Biospecimens and Genotyping

Germline DNA was extracted from saliva (Oragene kits, DNA Genotek, Inc.), PCR-amplified, and sequenced at the 951 bp one-exon region of *MC1R*, using standard procedures. Observed *MC1R* variants (Supplementary Table S1) were classified as higher risk based on elevated odds (OR ≥ 1.80) of melanoma, SCC, or BCC (10, 13) or having an HVAR score > 0.909 as computed by Polyphen (Polymorphism Phenotyping, RRID:SCR_013189; ref. 17). We classified participants as higher-risk if they carried at least one higher-risk variant, otherwise participants were classified as average risk.

### Randomization and Mailed Prevention Materials

Participants chose between English or Spanish language study materials. Participants who returned the baseline questionnaire and were successfully genotyped at *MC1R* (*n* = 920) were block-randomized within *MC1R* risk groups in 1:1 allocation ratio into precision prevention or standard arms, with each block having a sequence of assigning two participants to each arm. Computer-generated sequences were used to randomize the sequence of each block. We used Moffitt Cancer Center's web-based application, Subject Registration and Randomization, for centralized subject registration and randomization.

A sample size of 400 participants (200 in each arm) corresponded to 80% power to detect intervention effects comparable with a Cohen *d* = 0.30, based on two-sided tests, alpha = 0.05, autocorrelation of 0.75 to account for repeated measures of participants over time, and 20% study attrition.

Precision prevention materials were based on those developed by Hay and colleagues (18, 19), which were written in plain language accessible to individuals with low health literacy and health numeracy. Mailed precision prevention materials included information about (i) skin cancer; (ii) genetic risk for skin cancer; (iii) the *MC1R* gene and its contribution to skin cancer development, the participant's *MC1R* risk group and how their genetics impact their skin cancer risk; (iv) guidelines for skin cancer prevention based on their genetics; and (v) a guide to self/partner skin examinations. Mailed standard arm materials included information about (i) skin cancer and (ii) generic skin cancer prevention behaviors and skin exam guidelines adapted from recommendations by the American Academy of Dermatology (AAD). All participants additionally received a refrigerator magnet summarizing primary prevention behaviors, and sun protection guidelines targeted at children that were adapted from AAD recommendations. We excluded participants (*n* = 31) for whom genotyping failed. These individuals were mailed generic skin cancer prevention materials and a $10 cash or gift card.

Telephone and/or email follow-ups were conducted within 2 weeks after mailing prevention materials. These communications were not intended to recapitulate or reiterate prevention materials, but to verify the receipt the prevention materials and address participant questions. After successful contact (74.2%) or three unsuccessful attempts, participants were mailed a summary of the prevention materials (all participants) and a reminder of their *MC1R* risk group (intervention arm). After successful phone or email follow-up, participants in Tampa were sent a $20 gift card, and another $20 gift card after returning the 9-month survey; participants in Ponce were given $10 cash after telephone or email follow-up, and $20 cash upon completing the 9-month survey.

### Study Assessments

Self-reported race and ethnicity were assessed in the screening questionnaire. The baseline questionnaire asked about the participants’ education, marital status, gender, paid or unpaid work outdoors, untanned skin color, health numeracy and literacy, and family history of melanoma, SCC, and BCC, and other cancers. To measure health literacy, participants were asked “How confident are you filling out medical forms by yourself?” (not at all, a little bit, somewhat, quite a bit, extremely; ref. 20). To measure health numeracy, participants were asked “In general, how easy or hard do you find it to understand medical statistics?” (very easy, easy, hard, very hard; ref. 21). The baseline questionnaire additionally assessed psychosocial measures such as recent worry and concern about skin cancer (Supplementary Table S2). A supplemental baseline questionnaire completed by 80% of participants collected information on pigmentation characteristics, cancer fatalism (22), and familism (ref. 23; Table 1; Supplementary Table S2).

**TABLE 1:** Baseline characteristics of the study population.

	*MC1R* average risk, *n* (%)			*MC1R* higher risk, *n* (%)		
Variable	Standard arm (*n* = 195)	Precision prevention arm (*n* = 195)	*P* ^a^	Standard arm (*n* = 262)	Precision prevention arm (*n* = 268)	*P* ^a^
***Demographics***						
**Location**			1.00			0.94
Puerto Rico	93 (47.7%)	93 (47.7%)		123 (46.9%)	124 (46.3%)	
Tampa	102 (52.3%)	102 (52.3%)		139 (53.1%)	144 (53.7%)	
**Race**			0.81			0.18
White	149 (76.4%)	152 (77.9%)		221 (84.4%)	213 (79.5%)	
Other	46 (23.6%)	43 (22.1%)		41 (15.6%)	55 (20.5%)	
**Hispanic identity**			0.67			0.67
Puerto Rican	138 (70.8%)	131 (67.2%)		172 (65.6%)	174 (64.9%)	
Central/South American but not Brazilian	17 (8.7%)	14 (7.2%)		36 (13.7%)	40 (14.9%)	
Cuban	16 (8.2%)	22 (11.3%)		21 (8.0%)	17 (6.3%)	
Mexican	7 (3.6%)	4 (2.1%)		21 (8.0%)	19 (7.1%)	
Dominican (Republic)	4 (2.1%)	8 (4.1%)		2 (0.8%)	7 (2.6%)	
Mixed (more than one selected)	6 (3.1%)	9 (4.6%)		8 (3.1%)	7 (2.6%)	
Other	7 (3.6%)	7 (3.6%)		2 (0.8%)	4 (1.5%)	
**Spanish language materials (Tampa only)**	24 (23.5%)	20 (19.6%)	0.61	43 (30.9%)	29 (20.1%)	0.05
**Age in years (mean, SD)**	47.1 (15.4)	45.9 (14.9)	0.78	44.9 (15.7)	44.0 (15.5)	
**Female**	138 (70.8)	134 (68.7)	0.80	193 (73.7%)	186 (69.4%)	0.31
**Marital status**			0.28			0.93
Single or never married	55 (28.2)	51 (26.2%)		76 (29.0%)	75 (28.0%)	
Married, domestic partnership, or civil union	98 (50.3%)	112 (57.4%)		136 (51.9%)	143 (53.4%)	
Divorced, separated, or widowed	40 (20.5%)	30 (15.4%)		49 (18.7%)	48 (17.9%)	
**Education**			0.23			0.99
Graduate degree or higher	31 (15.9%)	18 (9.2%)		44 (16.8%)	40 (14.9%)	
Four-year college degree	30 (15.4%)	29 (14.9%)		40 (15.3%)	45 (16.8%)	
Some college^b^	49 (25.1%)	57 (29.2%)		68 (26.0%)	73 (27.2%)	
High school or GED	57 (29.2%)	63 (32.3%)		68 (26.0%)	61 (22.8%)	
Less than high school or GED	26 (13.3%)	26 (13.3%)		39 (14.9%)	42 (15.7%)	
**Family history of melanoma**	22 (11.3%)	26 (13.3%)	0.64	27 (10.3%)	36 (13.4%)	0.30
**Family history of skin cancer**	10 (5.1%)	10 (5.1%)	1.00	15 (5.7%)	12 (4.5%)	0.67
**Family history of other cancers**	108 (55.4%)	112 (57.4%)	0.76	143 (54.6%)	140 (52.2%)	0.75
**Worked outdoors**	70 (35.9%)	70 (35.9%)	1.00	97 (37.0%)	104 (38.8%)	0.71
**Health literacy**			0.66			0.15
Extremely confident	107 (54.9%)	99 (50.8%)		116 (44.3%)	136 (50.7%)	
Quite a bit confident	56 (28.7%)	65 (33.3%)		92 (35.1%)	78 (29.1%)	
Not at all, a little bit, or somewhat confident	32 (16.4%)	29 (14.9%)		53 (20.2%)	49 (18.3%)	
**Health numeracy**			0.53			0.02
Very easy	61 (31.3%)	52 (26.7%)		53 (20.2%)	76 (28.4%)	
Easy	98 (50.3%)	106 (54.4%)		150 (57.3%)	144 (53.7%)	
Hard or very hard	36 (18.5%)	35 (17.9%)		57 (21.8%)	45 (16.8%)	

^a^ *P* values are from *t* tests for normally distributed variables, Wilcoxon rank-sum tests for ordinal and non-normally distributed variables, and *χ*^2^ tests for categorical variables.

^b^Participants who indicated they were educated outside the United States were assigned to the median value (some college).

At baseline and after 3 and 9 months, participants reported on the following outcomes by answering a standardized survey of sun exposure and sun protection behaviors (24): (i) time spent outside (in hours) from 10 a.m. to 4 p.m. on weekdays and weekends separately; (ii) number of sunburns; (iii) frequency of outdoor intentional tanning (never, rarely, sometimes, often, always); (iv) number of tanning bed sessions; (v) frequency (never, rarely, sometimes, often, always) of each of the following sun protection behaviors: wearing of (i) hats, (ii) sunglasses, and (iii) shirts that cover the shoulders; (iv) parasol usage or standing in the shade while outdoors, and (v) sunscreen usage; (vi) total body skin examination (TBSE; yes/no) performed by a health provider; and (vii) number of self/partner skin examinations (SSE). A 3-item adaptation of the Lerman cancer worry scale was used to measure skin cancer worry (25, 26).

For participants who failed to return the 3-month survey, their 9-month survey was framed to measure outcomes since baseline. For participants whose 9-month survey due date was after March 15, 2020, that is, the start of lockdown activities for the SARS-CoV-2 pandemic, an additional 1-month grace period was provided to return surveys, and an indicator variable flagged those participants who experienced over 2 months of observation time during the lockdown.

Participants with at least one child ages 10 years of age or younger completed additional questions about the child's sex, age, untanned skin color, and primary prevention activities. Information on indoor tanning was not collected on children.

### Statistical Analysis

All analyses were conducted separately within the average- and higher-risk categories using SAS (Statistical Analysis System, RRID:SCR_008567). To assess the efficacy of the precision prevention intervention on the 3- and 9-month outcome measures and to estimate baseline and post-intervention population predicted marginal means of each outcome, we used generalized estimating equations (GEE). GEE models were adjusted for randomization imbalances, predictors of missingness, and predictors of each outcome. A type III *P* ≤ 0.05 was considered statistically significant. Type III *P* values test deviations from the null of a composite of point estimates precluding the estimation of 95% confidence intervals (CI) of the effect estimates.

Randomization was assessed by univariate comparisons of baseline variables between the intervention and standard arms; and variables showing a significant (*P* ≤ 0.05) imbalance were included as a covariate in statistical models. Univariate logistic regression analyses were used to identify baseline (and pandemic) predictors of missingness (*P* ≤ 0.05) separately for the 3- and 9-month assessments, followed by a backward stepwise selection by Akaike information criterion (ref. 27; Supplementary Table S3). A similar algorithm was used to identify baseline predictors of each outcome at each assessment. Main analyses included predictors from the main baseline questionnaire, and additional analyses were conducted to include predictors from the supplemental baseline questionnaire in the subset of participants who completed it.

Sun exposure hours (weekday and weekend), frequency of outdoor intentional tanning, number of sunburns, and cancer worry were modeled using the canonical identity link function, assuming a normal distribution of each outcome. The five sun protection behaviors were analyzed individually as repeated binary outcomes (often or always vs. sometimes, rarely, or never), and a logit link function was used. The low prevalence of indoor tanning (2.4%) precluded formal modeling of this outcome.

Child outcomes were modeled similarly, but due to the low number of participants with children (*N* = 125, 13.6%), some or all covariates were dropped. Because of sparse data on sun protection behaviors among children, we calculated a composite score for sun protection behaviors, which was equal to the number of behaviors practiced often or always. This variable was assumed to have a normal distribution.

Skin examination outcomes were dichotomized (ever vs. never having a skin examination during the study). TBSE and SSE were analyzed separately. ORs and 95% CIs were estimated using logistic regression. Participants (*n* = 6) who underwent a TBSE and those who underwent either a TBSE or SSE (*n* = 62) between screening and baseline were excluded from analysis of TBSE and SSE, respectively. Participants who did not return the 9-month survey were excluded from these analyses, and missingness predictors were not included in the model.

Baseline characteristics were evaluated as prospective moderators by individually testing the interaction term between the moderator and the study arm.

### Data Availability

The data will be shared upon reasonable request to the corresponding author.

### Role of the Funder

The funders had no role in the design of the study; in the collection, analyses, or interpretation of data; in the writing of the article, or in the decision to publish the results.

## Results

**FIGURE 1:**
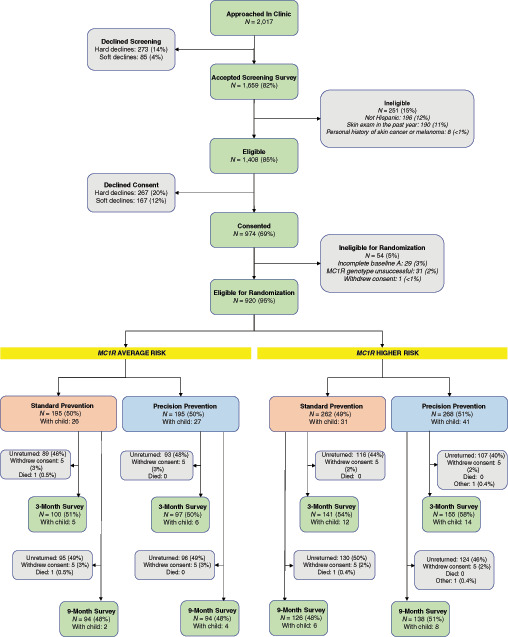
CONSORT diagram of the parallel randomized intervention trial.

Of the 1,408 (85%) individuals who were eligible for the study, 920 (65%) completed a baseline questionnaire and were randomized on the basis of their *MC1R* risk category (Fig. 1). Most participants reported sole Puerto Rican ethnic identity (67%) followed by Central or South American but not Brazilian identity (11.6%), and sole Cuban (8%) identity. The majority (80%) self-identified as white (Table 1). There were minimal differences in baseline characteristics by study arm (Table 1).

### *MC1R* Average-Risk Participants

Of the 390 study participants at *MC1R* average risk, 232 (59.5%) completed at least one follow-up questionnaire and could contribute to analyses of the precision prevention intervention. A total of 197 (51%) completed the 3-month survey and 188 (48%) completed the 9-month survey (Fig. 1); 153 participants (39.2%) returned both follow-up surveys.

There were significant reductions in weekday and weekend sun exposure hours, sunburns, and outdoor intentional tanning within each arm (*P* < 0.05; Table 2). However, there was no intervention effect on any primary prevention outcome (Table 2) or on skin examinations (Table 3).

**TABLE 2:** Primary prevention outcome measures at baseline and post-intervention and intervention effects by *MC1R* risk category.

	Standard arm			Precision prevention arm			Intervention effect^a^	
Outcome	Baseline	Post-intervention^b^	*P* ^c^	Baseline	Post-intervention^b^	*P* ^c^	Beta/OR	Type III *P*
	***MC1R* average risk**							
	***n* = 195**			***n* = 195**				
**Continuous outcomes^d^**								
Weekday sun exposure (hours)	1.70	1.38	0.005	1.60	1.28	0.01	−0.08	0.37
Weekend sun exposure (hours)	2.04	1.57	0.0003	1.88	1.62	0.02	0.15	0.67
Number of sunburns	0.42	0.10	<0.0001	0.52	0.12	<0.0001	0.09	0.18
Outdoor intentional tanning frequency	1.64	1.48	0.003	1.75	1.49	0.0005	−0.06	0.99
**Binary outcomes^e^**								
Wearing a hat often or always	0.15	0.19	0.26	0.12	0.17	0.12	0.94	0.87
Seeking shade or using umbrella often or always	0.42	0.52	0.02	0.43	0.46	0.54	0.72	0.20
Wearing a shirt with sleeves often or always	0.66	0.65	0.83	0.65	0.63	0.69	0.88	0.62
Wearing sunglasses often or always	0.52	0.52	0.85	0.46	0.49	0.51	1.07	0.86
Wearing sunscreen often or always	0.24	0.25	0.82	0.16	0.19	0.41	0.90	0.76
Indoor intentional tanning	0.02	0.02	—	0.03	0.01	—	—	—
	***MC1R* higher risk**							
	***n* = 262**			***n* = 268**				
**Continuous outcomes^d^**								
Weekday sun exposure (hours)	1.74	1.38	0.001	1.65	1.45	0.08	−0.05	0.99
Weekend sun exposure (hours)	2.05	1.67	<0.0001	1.95	1.57	<0.0001	−0.23	0.17
Number of sunburns	0.47	0.21	<0.0001	0.56	0.21	<0.0001	−0.12	0.19
Outdoor intentional tanning frequency	1.64	1.49	<0.0001	1.66	1.48	<0.0001	−0.04	0.41
**Binary outcomes^e^**								
Wearing a hat often or always	0.25	0.32	0.06	0.17	0.23	0.03	0.69	0.30
Seeking shade or using umbrella often or always	0.38	0.53	0.03	0.35	0.50	0.02	1.22	0.34
Wearing a shirt with sleeves often or always	0.67	0.68	0.61	0.70	0.72	0.78	0.83	0.44
Wearing sunglasses often or always	0.54	0.54	0.97	0.51	0.66	0.17	1.21	0.51
Wearing sunscreen often or always	0.13	0.13	0.96	0.15	0.24	0.08	1.74	0.03
Indoor intentional tanning	0.02	0.02	—	0.03	0.02	—	—	—

^a^Intervention effect compares the post-intervention measure in the intervention arm to that in the standard arm, after adjusting for baseline outcome, season, predictors of missingness, and predictors of the outcome.

^b^Post-Intervention is the average of outcome measures obtained at the 3- and 9-month assessments.

^c^Within arm *P* values are from tests comparing post-intervention measures to baseline averages from a GEE model containing baseline, 3- and 9-month outcomes as the dependent variables.

^d^Baseline and post-intervention values are population predicted marginal means, while the intervention effects are beta-coefficients.

^e^Baseline and post-intervention values are population predicted marginal proportions, while the intervention effects are reported as odds ratios (ORs). For indoor intentional tanning, only raw (unadjusted) proportions of participants who underwent indoor tanning are reported.

**TABLE 3:** Secondary prevention outcomes intervention effects by *MC1R* risk category.

	Intervention effect^a^			
Skin exam type^b^	OR	95% confidence interval		*P*
***MC1R* average risk**				
TBSE by health professional	1.54	0.57	4.18	0.40
SSE	0.65	0.33	1.31	0.23
***MC1R* higher risk**				
TBSE by health professional	6.51	2.23	19.02	0.0006
SSE	1.66	0.86	3.20	0.13

^a^Intervention effect compares the post-intervention measure in the intervention arm to that in the standard arm, after adjusting for season and baseline predictors of outcome.

^b^Participants who reported having had a TBSE completed by a health professional at the baseline assessment were excluded from analyses of TBSE health professional skin exams; and those who reported having completed a self/partner skin examination (SSE) or a TBSE by a health professional at the baseline assessment were excluded from analyses of SSE.

Familism significantly moderated the intervention effect on weekend sun exposure hours (*P* = 0.01; Table 4). The intervention resulted in fewer exposure hours (β = −0.39, *P* = 0.03) among participants with a familism score one SD below the mean but more exposure hours (β = 0.33, *P* = 0.06) among those who were one SD above. Location moderated the intervention effect on wearing sunglasses (*P* = 0.02). The intervention effect on sunglass use was positive (OR = 2.17, *P* = 0.10) among participants in Tampa and negative (OR = 0.36, *P* = 0.09) among Puerto Ricans. Natural hair color moderated the intervention effect on sunscreen use (*P* = 0.002). The intervention effect was negative (OR = 0.44, *P* = 0.07) among redheads/blondes and positive (OR, 5.04; *P* = 0.02) among brown/black-haired participants. Finally, age moderated the intervention effect on TBSE (*P* = 0.02), with an inverse association (OR = 0.86, *P* = 0.83) among participants at one SD above the mean age and a positive association (OR = 15.63, *P* = 0.03) for those at one SD below.

**TABLE 4:** Stratum-specific intervention effects for statistically significant moderators.

Outcome	Moderator	*P*_interaction_	Strata of moderator	Effect^b^	*P*
***MC1R* average risk**					
Weekend hours	Familism^a^	0.01	Mean + SD	0.33	0.06
			Mean − SD	−0.39	0.03
Wearing sunglasses	Location	0.02	Tampa	2.17	0.10
			Puerto Rico	0.36	0.09
Wearing sunscreen	Hair color^a^	0.002	Red or blonde	0.44	0.07
			Brown or black	5.04	0.02
TBSE by health professional	Age	0.02	Mean + SD	0.86	0.83
			Mean − SD	15.63	0.03
***MC1R* higher risk**					
Self or partner skin exam	Familism^a^	0.03	Mean + SD	5.77	0.01
			Mean − SD	1.02	0.96

^a^Variable was captured on the supplemental baseline questionnaire and moderation analyses included supplemental baseline variables as covariate predictors of missingness or outcome.

^b^Effects are shown as beta-coefficients for weekend hours, and as odds ratios for all other outcomes.

### *MC1R* Higher-Risk Participants

Of the 530 study participants at *MC1R* higher risk, 347 (65.5%) completed at least one follow-up questionnaire and could contribute to analyses of the precision prevention intervention. A total of 296 (56%) completed the 3-month survey and 264 (50%) completed the 9-month survey (Fig. 1); 213 participants (40.2%) returned both follow-up surveys.

When comparing baseline and post-intervention measures, we found a reduction in weekend sun exposure hours, sunburns, and outdoor intentional tanning within each arm, and a reduction in weekday hours in the standard group only (*P* < 0.05; Table 2).

The intervention group was more likely than the standard group to use sunscreen often or always (OR = 1.74, *P* = 0.03; Table 2) and was more likely to have completed a TBSE (OR = 6.51, *P* = 0.0006). Familism moderated the intervention effect on completing a SSE (*P* = 0.03; Table 4). The intervention effect was positive among participants at one SD above the mean familism score (OR = 5.77, *P* = 0.01) and null for those one SD below the mean (OR = 1.02, *P* = 0.96).

### Skin Cancer Worry

There was no intervention effect on skin cancer worry in either *MC1R* average-risk (*P* = 0.59) or higher-risk (*P* = 0.62) participants, and no changes in skin cancer worry between baseline and post-intervention within each arm (all *P* > 0.05; Supplementary Table S4).

### Outcomes Among Children

Among children of *MC1R* average-risk participants, there were no significant intervention effects on any outcome. There were reductions in weekend sun exposure hours (*P* = 0.0006) and outdoor intentional tanning (*P* = 0.01) in the intervention group only (Table 5).

**TABLE 5:** Primary prevention outcome measures among children of participants at baseline and post-intervention and intervention effects by the parent's *MC1R* risk category.

	Standard arm			Precision prevention arm			Intervention effect^a^	
Outcome	Baseline	Post-intervention^b^	*P* ^c^	Baseline	Post-intervention^b^	*P* ^c^	Beta/OR	Type III *P*
	***MC1R* average risk**							
	***n* = 26**			***n* = 27**				
**Continuous outcomes^d^**								
Weekday hours	1.48	1.33	0.09	1.49	1.04	0.12	0.34	0.43
Weekend hours	1.80	1.56	0.18	2.18	0.44	0.0006	−1.08	0.38
Sun protection behaviors	1.44	1.21	0.82	1.51	1.68	0.48	−0.51	0.42
Sunburns	0.53	0.53	0.13	0.54	−0.44	0.14	0.04	0.70
Outdoor intentional tanning	1.43	0.93	0.15	1.39	1.64	0.01	0.35	0.14
**Binary outcomes^e^**								
Wearing a hat often or always^f^	0.04	0.00	—	0.04	0.00	—	—	—
Seeking shade or using umbrella often or always^g^	0.16	0.37	0.21	0.30	0.46	0.33	2.27	0.70
Wearing a shirt with sleeves often or always^f^	0.65	1.00	—	0.71	0.71	—	—	—
Wearing sunglasses often or always^f^	0.04	0.00	—	0.07	0.08	—	—	—
Wearing sunscreen often or always^f^	0.31	0.20	—	0.32	0.27	—	—	—
	***MC1R* higher risk**							
	***n* = 31**			***n* = 41**				
**Continuous outcomes^d^**								
Weekday hours	1.73	2.46	0.08	1.53	0.90	0.02	−0.94	0.005
Weekend hours^h^	1.87	2.18	0.32	1.88	0.84	0.02	−1.15	0.05
Sun Protection behaviors^h^	1.50	1.13	0.03	1.55	1.98	0.16	0.93	0.02
Sunburns	0.29	1.21	0.13	0.20	−0.07	0.04	−0.55	0.06
Outdoor intentional tanning^h^	1.24	1.35	0.41	1.22	1.06	0.23	0.12	0.34
**Binary outcomes^e^**								
Wearing a hat often or always^f^	0.06	0.08	—	0.17	6.36	—	—	—
Seeking shade or using umbrella often or always	0.32	0.52	0.66	0.32	0.46	0.32	1.22	0.87
Wearing a shirt with sleeves often or always^g^	0.61	0.62	0.86	0.68	0.69	0.96	1.06	0.94
Wearing sunglasses often or always^f^	0.06	0.00	—	0.05	0.17	—	—	—
Wearing sunscreen often or always^i^	0.32	0.29	0.79	0.39	0.36	0.80	2.38	0.41

^a^Intervention effect compares the post-intervention measure in the intervention arm to that in the standard arm, after adjusting for baseline outcome, season, predictors of missingness, and predictors of the outcome.

^b^Post-intervention is the average of outcome measures obtained at the 3- and 9-month assessments.

^c^Within arm *P* values are from tests comparing post-intervention measures to baseline averages from a GEE model containing baseline, 3- and 9-month outcomes as the dependent variables.

^d^Baseline and post-intervention values are population predicted marginal means, while the intervention effects are beta-coefficients.

^e^Baseline and post-intervention values are population predicted marginal proportions, while the intervention effects are reported as ORs.

^f^Because of sparse data, raw proportions are presented.

^g^Because of sparse data, unadjusted values are presented.

^h^Because of sparse data, within-arm estimates were modeled without missingness predictors.

^i^Because of sparse data, all estimates were modeled without missingness predictors.

Among children of *MC1R* higher-risk participants, there were two significant intervention effects: weekday sun exposure hours decreased (β = −0.94, *P* = 0.005) and the number of sun protection behaviors practiced often or always increased (β = 0.93, *P* = 0.02). The intervention group exhibited reductions in weekday (*P* = 0.02), weekend (*P* = 0.02) sun exposure hours, and sunburns (*P* = 0.04). The standard group exhibited a reduction in sun protection behaviors (*P* = 0.03).

## Discussion

In this randomized trial of *MC1R* precision prevention materials, we observed significant intervention effects on sunscreen use and TBSE among *MC1R* higher-risk individuals. In addition, we observed significant improvements in weekday sun exposures and sun protection behaviors among their children. In contrast, we observed no intervention effects among *MC1R* average-risk individuals or their children, and improved preventive behaviors within both the standard and intervention arms, dispelling concerns that provision of low to average genetic risk would give a false sense of security and increase risky behavior (28). We also report the absence of intervention effects on cancer worry in either *MC1R* higher- or average-risk participants, and no changes in cancer worry between baseline and post-intervention, indicating that our intervention did not harm participants’ psychological well-being. These findings support our hypotheses that feedback of precision prevention information would be actionable only among individuals who were informed of their higher risk for skin cancer.

Our study advances the evidence supporting a low intensity public health genomic approach to increase skin cancer preventive behaviors among Hispanics, a population underserved in precision medicine (29). Our findings are consistent with a systematic review and meta-analysis of 17 studies on the behavioral impact of genetic testing for complex diseases that reported significant increases in self-reported behavior change after providing results among risk variant carriers compared with non-carriers (30), and with results from our randomized controlled trial among non-Hispanic White individuals with limited melanoma risk phenotypes that found *MC1R* precision prevention materials improved some melanoma prevention activities (31).

Given that U.S. Hispanics do not engage routinely in sun protection behavior (32), including wearing sunscreen (33) or undergoing TBSE (34), our precision prevention findings are notable. Despite the intervention effect on sunscreen use, the population predicted marginal mean proportion of higher-risk individuals who often or always used sunscreen at post-intervention remained suboptimal at 24% (Table 2), and the overall unadjusted proportion at 9 months was 30%. In addition, our findings on skin examinations were based on small numbers—only 18 (10%) *MC1R* average-risk and 29 (11%) higher-risk participants who returned their 9-month follow-ups reported ever having a TBSE during follow-up—consistent with the 11% of screened individuals who were ineligible for the study because they had a TBSE in the previous year (Fig. 1). Thus, there is a need for more general skin cancer and sun protection awareness campaigns targeted to Hispanics.

Despite small numbers and without explicitly stating *MC1R* risk may be inherited by children, we observed significant intervention effects on some primary preventive activities among children of higher-risk participants. Childhood and adolescence are critical windows of exposure for skin cancer (35), and sun-seeking behaviors and tanning preferences among parents are reflected in their children (36). Thus, precision intervention may have cross-generational effects that can reduce skin cancer burden in subsequent generations, especially among Hispanic children, who have high rates of sunburn (37).

Higher familism was associated with increased likelihood to undergo self or partner skin exams among higher-risk intervention participants. Although studies of the effect of familism on disease screening among Hispanics are sparse, familism and the use of familism-based messaging has been shown to increase cancer screening among Hispanic women (38–40). Further research is warranted on how familism influences tendency for disease screening.

We found unexpected intervention effects in subgroups of *MC1R* average-risk participants. Some of these moderation effects are concerning, such as increased weekend sun exposure with increasing familism, decreased sunscreen use among red/blonde-haired participants, and decreased likelihood of TBSE with increasing age. Further investigations are needed to elucidate mediating factors and further emphasize that *MC1R* average risk does not equate to absence of risk.

Hispanics are underserved in health care (41) and are often difficult to recruit and retain in studies (42). Despite our 69% consent rate (Fig. 1), having bilingual/bicultural staff, and providing Spanish language materials as recommended by previous studies (43), only 54% and 49% of participants returned the 3- and 9-month questionnaire, respectively. Participants enrolled at baseline who did not complete at least one follow-up survey were more likely to be younger, male, non-White, have lower levels of education and health literacy, have decreased belief that skin cancer prevention activities are effective, and have decreased perceived skin cancer risk and skin cancer worry, although specific predictors of questionnaire missingness differed according to *MC1R* risk category and follow-up timepoint (Supplementary Table S3). Overall, the degree of missingness of participant post-intervention outcome measures serves to limit the generalizability of our results.

In addition, Hispanic men were underrepresented (28% of randomized participants) in our study, which may limit the generalizability of our results to this population. Hispanic men are less likely to seek medical care than Hispanic women (41, 44), which we observed at our Morsani (Tampa) recruitment clinics—only 37% of Hispanic patients with scheduled appointments during our recruitment period were male. Moreover, survey completion rates were lower among enrolled male participants; 47% and 40% returned the 3- and 9-month questionnaires, respectively.

In conclusion, provision of skin cancer precision prevention materials based on *MC1R* risk group can improve sunscreen use and tendency to undergo a TBSE among *MC1R* higher-risk Hispanics and may improve some primary prevention activities among their children. However, our study also reveals suboptimal skin cancer prevention behavior among Hispanics, necessitating targeted prevention campaigns. Finally, future research in Hispanics may need to consider strategies to increase recruitment and retention, particularly for Hispanic men.

## Supplementary Material

Supplementary Data Supplementary Tables 1, 2, 3, and 4.
